# MiR‐634 sensitizes glioma cells to temozolomide by targeting CYR61 through Raf‐ERK signaling pathway

**DOI:** 10.1002/cam4.1351

**Published:** 2018-02-23

**Authors:** Zhigang Tan, Jizong Zhao, Yugang Jiang

**Affiliations:** ^1^ Department of Neurosurgery The Second Xiangya Hospital of Central South University Changsha 410011 China; ^2^ Department of Neurosurgery Tiantan Hospital Capital Medical University Beijing 100050 China

**Keywords:** Drug resistance, glioma, microRNA, signaling, temozolomide

## Abstract

Glioma is the most common intracranial malignant tumors, accounting for about 40% of intracranial tumors. Primary or secondary drug resistance is one of the main reasons for the failure of treatment. The oncogenic or tumor‐suppressive roles of miR‐634 have been revealed in different types of cancer. However, the role of miR‐634 in glioma remains unknown and whether miR‐634 could sensitize glioma cells to temozolomide also is unclear. Here, we aim to investigate the biological function of miR‐634 and the possible mechanisms in glioma. In this study, we found that miR‐634 was downregulated in glioma tissues compared with normal brain tissues, and its expression was associated with tumor size and WHO grade. Importantly, glioma patients with low miR‐634 expression showed a shorter survival time than patients which had high expression of miR‐634. This study also showed that miR‐634 was decreased in temozolomide‐resistant glioma cells, and restoration of miR‐634 could sensitize the resistant cells to temozolomide by targeting CYR61 through Raf‐ERK signaling. Our study provides a potential target for overcome drug resistance in glioma.

## Introduction

Glioma is the most common intracranial malignant tumors, accounting for about 40% of intracranial tumors 1. Glioma has high degree of malignancy, rapid growth, short duration, easy to relapse after surgery, poor prognosis, and other characteristics, which is considered to be one of the most difficult surgical fields of neurosurgery 2, 3. The exact etiology of glioma is not clear. The occurrence and progression of glioma are closely related with a variety of overexpressed oncogenes and inactivated antioncogenes 4. It is important to further explore the relationship between the aberrant expression of related genes and the progress of glioma and its malignant features, which may help us to reveal the precise mechanism of tumorigenesis, early diagnosis, and treatment of glioma 5.

The main treatment of glioma is to take operation, combined with chemotherapy and radiotherapy 6. Primary or secondary drug resistance is one of the main reasons for the failure of treatment. The effective rate of commonly used drugs such as cisplatin, carmustine, and teniposide is only about 20%, and the efficacy of the latest chemotherapy drugs temozolomide is only 35% 7, 8. Multidrug resistance (MDR) of tumor cells is more likely to result tumor chemotherapy failure 9. Therefore, overcoming chemoresistance is especially crucial for improving the survival rate of glioma patients.

MicroRNAs are a class of small noncoding RNAs. They play important roles on cell proliferation, migration, and other biological processes by degrading their targets. The oncogenic or tumor‐suppressive roles of miRNAs have been revealed in different types of cancer 10, 11, 12, 13. MiR‐634 is located in intron 15 of protein kinase C alpha (PRKCA) gene, which is a Pol III‐dependent intronic miRNA, which could target its host gene through a “first‐order” negative feedback 14. Comprehensive microarray‐based analysis of the miRNome of human epidermoid carcinoma cells revealed an up to 15‐fold transient overexpression of miR‐634 after photodynamic therapy (PDT) 15. Recent studies have revealed that miR‐634 is a sensitizer to chemotherapy in several cancers, including hepatocellular carcinoma 16, ovarian cancer 17, esophageal squamous cell carcinoma 18, and nasopharyngeal carcinoma 19. These suggest that miR‐634 is a critical target for overcoming drug resistance. However, the role of miR‐634 in glioma remains unknown and whether miR‐634 could sensitize glioma cells to temozolomide is also unclear. Here, we aim to investigate the biological function of miR‐634 in glioma and the possible mechanisms.

## Materials and Methods

### Study population

Forty‐six glioma tissue samples were collected from The Second Xiangya Hospital from 17 January 2012 to 18 December 2016, including 26 males and 20 females, aged 32–70 years with an average age of 50. These cases were histopathologically confirmed and have follow‐up data. All patients received no radiotherapy and chemotherapy before surgery. The samples were divided into I–IV level according to the 2007 edition of the fourth edition of WHO central nervous system tumor grading criteria, including four cases grade I, 11 cases grade II, 14 cases grade III, and 17 cases grade IV. There are also 10 cases of normal brain tissue from brain trauma as a normal control. The study was approved by the Ethics Committee of The Second Xiangya Hospital of Central South University. All the participants involved in this study have signed the informed consent.

### Cell culture

Human glioma cell line U251 and U87 were obtained from the cancer hospital at the Chinese Academy of Medical Sciences (Beijing, China). Cells were cultured with RPMI1640 containing 10% fetal bovine serum (FBS, Gibco‐BRL, Carlsbad, CA, USA) and maintained in a humidified at atmosphere of 5% CO_2_ in air at 37°C. The temozolomide (TMZ)‐resistant glioma cell line was established as Tian et al. described 20. The U251 cells (1 × 10^5^/mL) were incubated for 24 h, and then, the initial concentration of temozolomide (5 μm) was added. The medium containing TMZ was changed once every 2–3 days. When the initial dose was induced for two weeks, the drug dose was doubled, and each dose was maintained for 2 weeks. The final concentration was increased to 400 μm. The U251 drug‐resistant cell line was named U251/TMZ. The method for induction U87 drug‐resistant cell line is consistent with U251/TMZ.

### Reagents

The following antibodies were used in this study: *β*‐actin (Santa Cruz Biotechnology, Dallas, TX, USA), GST‐*π*, P‐gp, MDR1, p‐Raf, Raf, p‐MEK, MEK p‐ERK, ERK, and CYR61 (Cell Signaling Tech, Danvers, MA, USA). U0126 was obtained from Selleck (Selleck, Shanghai, China). Temozolomide was obtained from Sigma‐Aldrich (St. Louis, MO, USA).

### Cell treatment

The lentivirus expression plasmid containing CYR61 and empty vector control were purchased from Genepharma Company (Shanghai, China). Cells were transfected with these lentivirus using Lip3000 (Life technologies, Carlsbad, CA, USA) according to the manufacturers’ instructions. Overexpression of miR‐634 was performed using miR‐634 mimic (GeneCopoecia, Guangzhou, China) The cells transfected with scramble control were used as negative control. Cells were plated in 6‐well clusters or 96‐well plates and transfected for 48 h. Transfected cells were used in further assays or protein extraction.

### RNA extraction and SYBR green quantitative PCR analysis

Total RNA was extracted from cells using TRIzol reagent (Invitrogen, Carlsbad, CA, USA). MiR‐634 expression in cells was detected using a Hairpin‐it TM miRNAs qPCR kit (Genepharma, Shanghai, China) according to manufacturers’ instructions. Expression of RNU6B was used as an endogenous control. QPCR was performed at the condition: 95.0°C for 3 min, and 40 circles of 95.0°C for 10 s and 60°C for 30 s. Data were processed using 2^−ΔΔCT^ method.

### Cell viability assay and colony formation assay

Twenty‐four hours before the experiment, cells were plated in 96‐well plates at a density of 1000 cells in 100 μL medium per well. The cells were treated with a range of different concentration of TMZ, and the cell viability was assessed by CKK‐8 assay (Beyotime, Shanghai, China) according to the manufacturers’ instructions.

For the colony formation assay, following treatment, adherent cells were trypsinized and 1000 viable cells were subcultured in six‐well plates (in triplicate). Cells were allowed to adhere and colonize for two weeks. To visualize colonies, media were removed and cells were fixed in 96% ethanol for 10 min and then stained with crystal violet staining solution. The colonies were captured and counted.

### Annexin V‐FITC staining and flow cytometry

Staining was performed using Annexin V‐FITC kit following the manufacturer's instructions (KeyGEN BioTech, Nanjing, China). Briefly, 2 × 10^5^ cells were harvested by centrifugation at 1000 g for 5 min and resuspended in 100 μL binding buffer, followed by 15 min incubation with 5 μL Annexin V‐FITC in the dark at 37°C. After that, 10 μL PI staining was added with gentle shaking for 10 min incubation in the dark at 37°C. Flow cytometry (BD, Frankin Lakes, NJ, USA) analysis was employed for detecting apoptotic events.

### Western blotting

Cells were lysed in cold RIPA buffer, and the protein was separated with 10% SDS‐PAGE, which was then transferred to PVDF membrane (Thermo Fisher, Waltham, MA, USA). After that, the membrane was incubated in PBS with 5% nonfat dried milk (Mengniu, Hohhot, China) for 3 h at 4°C. Then, the membrane was incubated with primary antibodies overnight at 4°C and then with appropriate secondary antibody (Abcam, Cambridge, UK) for 1 h at 37°C. The immune complexes were detected using ECL Western Blotting Kit (Millipore, Boston, MA, USA). The relative protein expression was analyzed using Image‐Pro plus software 6.0 (Media Cybernetics Inc, Rockville, MD), and *β*‐actin was used as the internal reference.

### Statistical analysis

In this study, all experiments were repeated at least three times, and all data are expressed as the mean ± SEM. SPSS 18.0 software package (SPSS, Chicago, IL, USA) was used to perform statistical analysis. Difference between two groups was compared by independent samples *t* test. Difference among three or more groups was compared by one‐way anova. The *P* value <0.05 was considered statistically significant.

## Results

### MiR‐634 is downregulated in glioma

**Figure 1:**
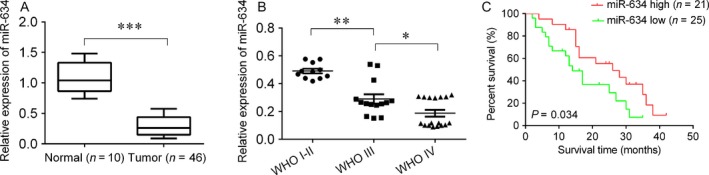
The expression of miR‐634 in glioma tissues. (A) QPCR was used to determine the expression of miR‐634 in glioma tissues and normal control. (B) The expression of miR‐634 in normal tissues and grade I+II and grade III+IV. (C) Survival rate analysis in glioma patients with low or high miR‐634 expression. *P < 0.05, **P < 0.01, ***P < 0.001.

**Figure 2:**
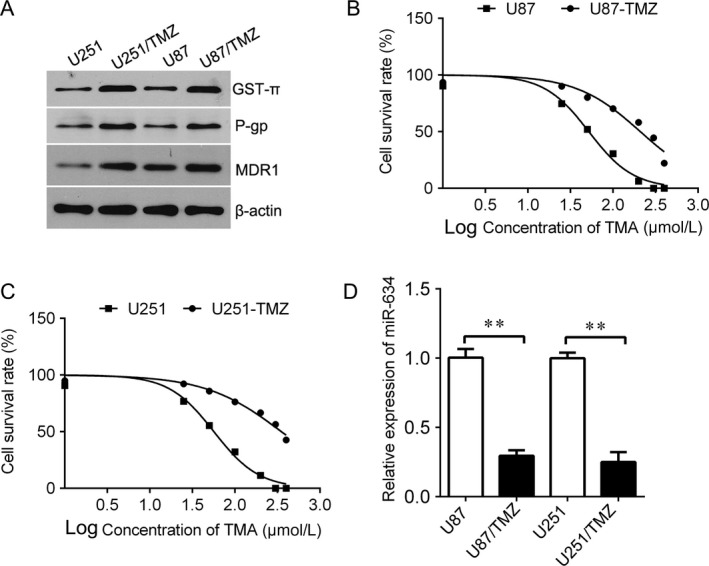
The expression of miR‐634 in temozolomide‐resistant glioma cells. (A) Western blot analysis for drug‐resistant related genes, GST‐*π*,P‐gp, and MRP, in U251/TMZ and U87/TMZ cells. (B) CKK‐8 assay was used to determine the cell viability in U87/TMZ. (C) CKK‐8 assay was used to determine the cell viability in U251/TMZ. (D) QPCR analysis for miR‐634 in U251/TMZ and U87/TMZ cells. TMZ, temozolomide. *P < 0.05, **P < 0.01, ***P < 0.001.

To investigate the role of miR‐634 in glioma, firstly, we evaluated the expression of miR‐634 in glioma tissues. We found that miR‐634 was significantly decreased in glioma tissues compared with normal control (Fig. 1A). Importantly, the expression of miR‐634 was lower in WHO grade III and IV than in WHO grade I and II (Fig. 1B), suggesting the expression of miR‐634 was relevant with glioma progress. We also found that miR‐634 expression was associated with tumor size, WHO grade, and KPS score (Table 1). Moreover, the glioma patients were divided into two groups according to the median of miR‐634. We found that the patients with low miR‐634 have shorter survival time than that of with high miR‐634 expression (Fig. 1C). And we also wanted to know the expression of miR‐634 in drug‐resistant glioma cells. The temozolomide‐resistant U251 and U87 cells were generated by increasingly treating temozolomide. As shown in Figure 2A, the drug‐resistant related genes, including GST‐*π*, P‐gp, and MRP, were significantly increased in U251/TMZ and U87/TMZ cells compared with the parental cells. And we treated the cells with a range of concentrations of TMZ to measure the half maximal inhibitory concentration (IC_50_). We found that the IC50 of parental cells was significantly lower than those of in U251/TMZ and U87/TMZ cells (Fig. 2B,C). Interestingly, the expression of miR‐634 was significantly decreased in U251/TMZ and U87/TMZ cells compared with the parental cells (Fig. 2D), indicating that downregulation of miR‐634 may be involved in glioma drug resistance.

**Table 1:** The association of miR‐634 and clinical features in glioma

Feature	Number	miR‐634 expression		*P* value
		Low (*n* = 25)	High (*n* = 21)	
Age (years)				
≥50	28	15	13	0.569
<50	18	10	8	
Gender				
Male	26	14	12	0.588
Female	20	11	9	
Tumor size (cm)				
≥5	25	10	15	0.042
<5	21	15	6	
WHO grade				
I+II	15	4	11	0.010
III+IV	31	21	10	
KPS score				
≥70	19	7	12	0.044
<70	27	18	9	

### Restoration of miR‐634 sensitizes glioma cells to temozolomide

**Figure 3:**
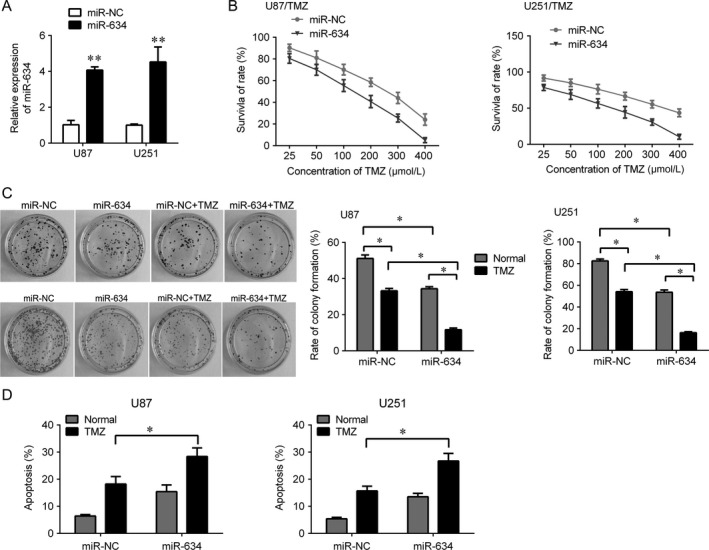
Restoration of miR‐634 overcomes temozolomide resistance in U251/TMZ and U87/TMZ cells. (A) QPCR analysis for miR‐634 in U251/TMZ and U87/TMZ cells after transfection with miR‐634 mimic or scramble control. (B) CKK‐8 assay was used to determine the cell viability after restoration of miR‐634. (C) Colony formation assay was used to determine the cell growth after restoration of miR‐634 and TMZ treatment. (D) Flow cytometry was used to measured cell apoptosis after restoration of miR‐634 and TMZ treatment. *P < 0.05, **P < 0.01.

We further investigated whether restoration of miR‐634 expression could relief the TMZ resistant in U251/TMZ and U87/TMZ cells. We upregulated the expression of miR‐634 in U251/TMZ and U87/TMZ cells (Fig. 3A). We observed that upregulation of miR‐634 significantly reduced the IC50 compared with control group (120 μm vs. 240 μm in U87/TMZ cells; 100 μm vs. 260 μm in U251/TMZ cells, Fig. 3B). Furthermore, we performed flow cytometry and colony assay to evaluate the role of miR‐634 on U251/TMZ and U87/TMZ cells apoptosis and proliferation. We found that overexpression of miR‐634 inhibited the cell proliferation and promoted apoptosis (Fig. 3 C,D). We also found that restoration of miR‐634 cells significantly sensitized U251/TMZ and U87/TMZ to TMZ evaluated by the significant reduce of colonies number (Fig. 3C) and the significant increase in apoptosis rate (Fig. 3D) compared with scramble control.

### MiR‐634 sensitizes glioma cells to temozolomide through CYR61 and Raf/MEK/ERK signaling

**Figure 4:**
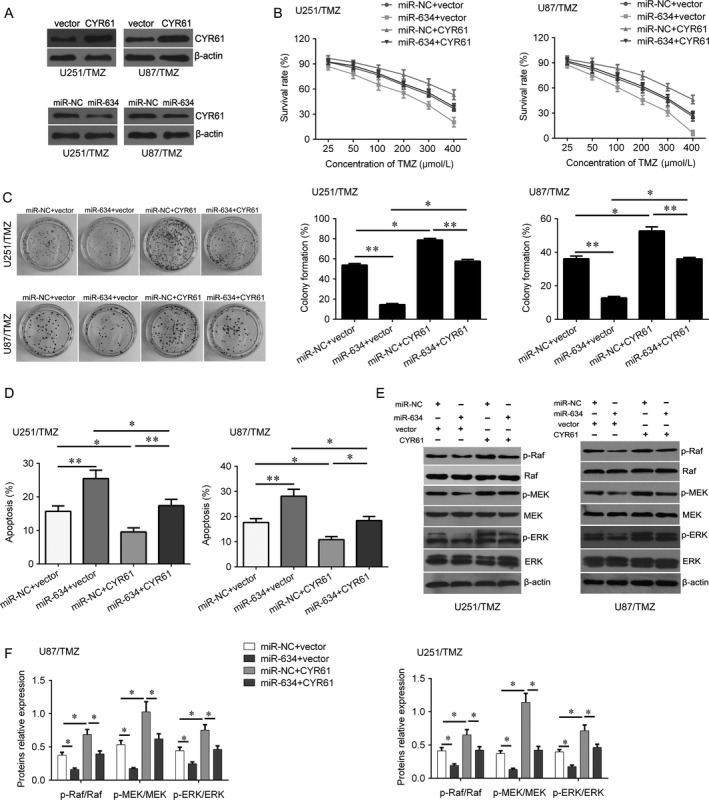
Overexpression of CYR61 reverses the inhibitory effects of miR‐634 downregulation in U251/TMZ and U87/TMZ cells. (A) Western blot analysis for CYR61 after transfection with CYR61 lentivirus plasmid or miR‐634 mimics. (B) CKK‐8 assay was used to determine the cell viability after restoration of miR‐634 and overexpression of CYR61. (C) Colony formation assay was used to determine the cell growth after restoration of miR‐634 and overexpression of CYR61. (D) Flow cytometry was used to measured cell apoptosis after restoration of miR‐634 and overexpression of CYR61. (E) Western blot analysis for p‐Raf, Raf, p‐MEK, MEK, p‐ERK, and ERK after restoration of miR‐634 and overexpression of CYR61. (F) Quantification of the Western blot bands in (E). *P < 0.05, **P < 0.01.

**Figure 5:**
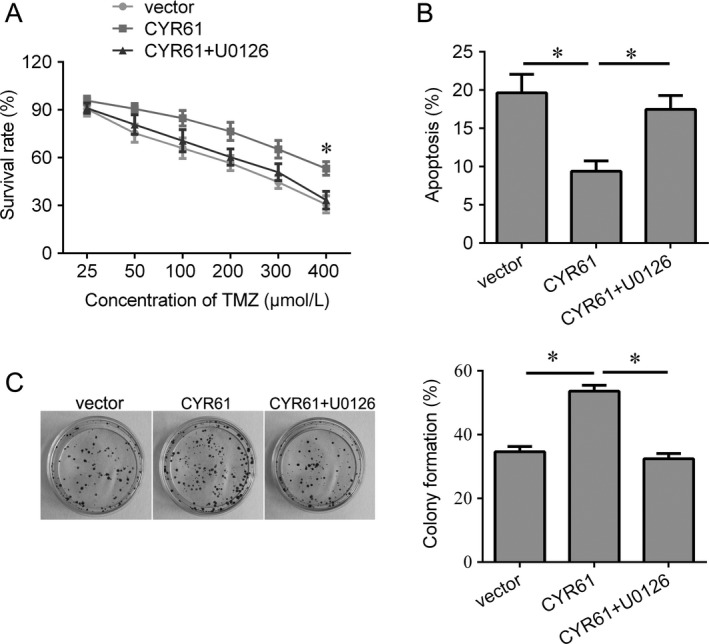
Inactivation of ERK signaling reverses the oncogenic effects of CYR61 in U87/TMZ cells. (A) CKK‐8 assay was used to determine the cell viability after CYR61 transfection and U0126 treatment. (B) Flow cytometry was used to measured cell apoptosis after CYR61 transfection and U0126 treatment. (C) Colony formation assay was used to determine the cell growth after CYR61 transfection and U0126 treatment. *P < 0.05.

Previous study showed that CYR61 was one of targets of miR‐634. Here, we evaluated whether miR‐634 sensitizes glioma cells to temozolomide through CYR61. We overexpressed miR‐634 and CYR61 in U251/TMZ and U87/TMZ cells (Fig. 4A). Induction of miR‐634 decreased the expression of CYR61, while transfected by CYR61 plasmid, the expression of CYR61 was increased (Fig. 4A). Overexpression of CYR61 increased the survival rate of U251/TMZ and U87/TMZ cells after TMZ treatment, while induction of miR‐634 significantly suppressed the survival of U251/TMZ and U87/TMZ cells after TMZ treatment (Fig. 4B). However, the inhibition induced by miR‐634 was abolished by CYR61 overexpression (Fig. 4B). Similar results were also found in colony formation assay and apoptosis assay (Fig. 4C,D). In addition, we found that restoration of miR‐634 could inactivate Raf/MEK/ERK signaling, whereas CYR61 activated this signaling (Fig. 4E). Importantly, upregulation of CYR61 reversed this inactivation of Raf/MEK/ERK signaling induced by miR‐634(Fig. 4E). Furthermore, we treated the U87/TMZ cells with U0126 to inactivate ERK signaling. As shown in Figure 5, the results demonstrated that inactivation of ERK signaling by U0126 can reverse the oncogenic effects of CYR61 evaluated by cell viability, apoptosis, and colony formation in U87/TMZ cells.

## Discussion

In this study, we found that miR‐634 was downregulated in glioma tissues compared with normal brain tissues, and its expression was associated with tumor size and WHO grade. Importantly, glioma patients with low miR‐634 expression showed a shorter survival time than that of with high miR‐634 expression, suggesting that miR‐634 may be a prognostic biomarker for glioma patients. Molecular markers redefine tumor subtypes within each grade of malignancy and providing diagnostic and prognostic information 21. It has been shown that miRNAs are integrally involved in brain gliomas’ genesis and progression 5. For example, patients with high miR‐1825 expression had a higher survival rate, and miR‐1825 expression levels were dependent on tumor size and pathological grading in glioma patients. MicroRNA‐Gene Ontology network indicated that miR‐1825 may play an important role in the progress of human glioma including apoptosis, cell proliferation, and invasion, which may be used as a biomarker for identification the pathological grade of glioma 22. MiR‐21 is one of the most studied miRNAs and is overexpressed in cancer tissues. Qu et al. 23 analyzed that extracellular miR‐21 exhibited an outstanding diagnostic accuracy in detecting brain cancer, and this accuracy was more obvious in glioma diagnosis. Thus, combination of several miRNAs may be useful for precious prediction of glioma genesis and progression.

Drug resistance to chemotherapy remains clinically problematic. The present study also showed that miR‐634 was decreased in temozolomide‐resistant glioma cells, and restoration of miR‐634 could sensitize the resistant cells to temozolomide. MiR‐634 also has been revealed as a sensitizer in other types cancer. MiR‐634 was significantly downregulated in the paclitaxel‐resistant nasopharyngeal carcinoma cells. Restoration of miR‐634 resensitized the nasopharyngeal carcinoma cells to paclitaxel in vitro and in vivo 19. Overexpression of miR‐634 activated the mitochondrial apoptotic pathway and enhanced chemotherapy‐induced cytotoxicity in a model of esophageal squamous cell carcinoma 18. However, miR‐634 can resensitize resistant ovarian cancer cell lines and patient‐derived drug‐resistant tumor cells to cisplatin, carboplatin, and doxorubicin, but not to paclitaxel in ovarian cancer cells 17. Our findings illustrate restoration of miR‐634 in resistant glioma cells may offer a broadly useful approach to overcome required drug resistance.

CYR61 is a target of miR‐634 in glioblastoma multiforme 20. We also confirmed this relationship in glioma cells. Overexpression of miR‐634 repressed CYR61 expression. And miR‐634‐mediated resensitization to temozolomide was attenuated by CYR61 overexpression. The CYR61 levels were significantly higher in glioblastoma than in the adjacent nontumor tissues. Overexpression of CYR61 enhanced the viability of glioblastoma cells through activating PI3K/Akt/mTor signaling pathway to increase cell growth in glioblastoma cells, while inhibition of CYR61 decreased the viability of glioblastoma cells in vitro and in vivo 24. Targeting CYR61 expression with siRNA inhibited hepatocyte growth factor‐induced cell migration and cell growth in vitro by inhibiting a second phase of Akt phosphorylation after cell stimulation with hepatocyte growth factor. Preestablished subcutaneous glioma xenografts with CYR61 siRNA inhibited glioma xenograft growth in a dose‐dependent manner 25. And CYR61 was also able to promote breast cancer cell proliferation, cell survival, and Taxol resistance through an alphavbeta3‐activated ERK1/ERK2 MAPK signaling 26 and to confer resistance to mitoxantrone via spleen tyrosine kinase activation in human acute myeloid leukemia 27. In line with our recent results that miR‐634 functions as tumor suppressor by targeting CYR61 through Raf‐ERK signaling, van Jaarsveld MT also found that the cell cycle regulator CCND1 and Ras‐MAPK pathway components GRB2, ERK2, and RSK2 were directly repressed by miR‐634 overexpression 17. Repression of the Raf‐MEK pathway using a MEK inhibitor abolished the CYR61 effects on cell proliferation and chemosensitivity. Additionally, miR‐634 may also via mTOR signaling to inhibit cell proliferation, migration, and invasiveness 28.

In summary, our studies demonstrated that downregulation of miR‐634 was associated with poor outcome of glioma patients. And restoration of miR‐634 can resensitize temozolomide‐resistant glioma cells to temozolomide through inactivating Raf‐ERK signaling by targeting CYR61. Our study provides a potential target for overcome drug resistance in glioma.

## Conflict of Interest

None declared.

## Supporting information


**Figure S1**. Overexpression of CYR61 reverses the inhibitory effects of miR‐634 downregulation on U87/TMZ cells.

**Figure S2**. Overexpression of CYR61 reverses the inhibitory effects of miR‐634 downregulation in U87 cells.

**Figure S3**. Inactivation of ERK signaling reverses the oncogenic effects of CYR61 in U87 cells.
