# Supplementary figures and images for: MiR‐634 sensitizes glioma cells to temozolomide by targeting CYR61 through Raf‐ERK signaling pathway
Source: Cancer Med. 2018 Feb 23;7(3):913–21. doi: 10.1002/cam4.1351 (PMC5852346; doi:10.1002/cam4.1351)

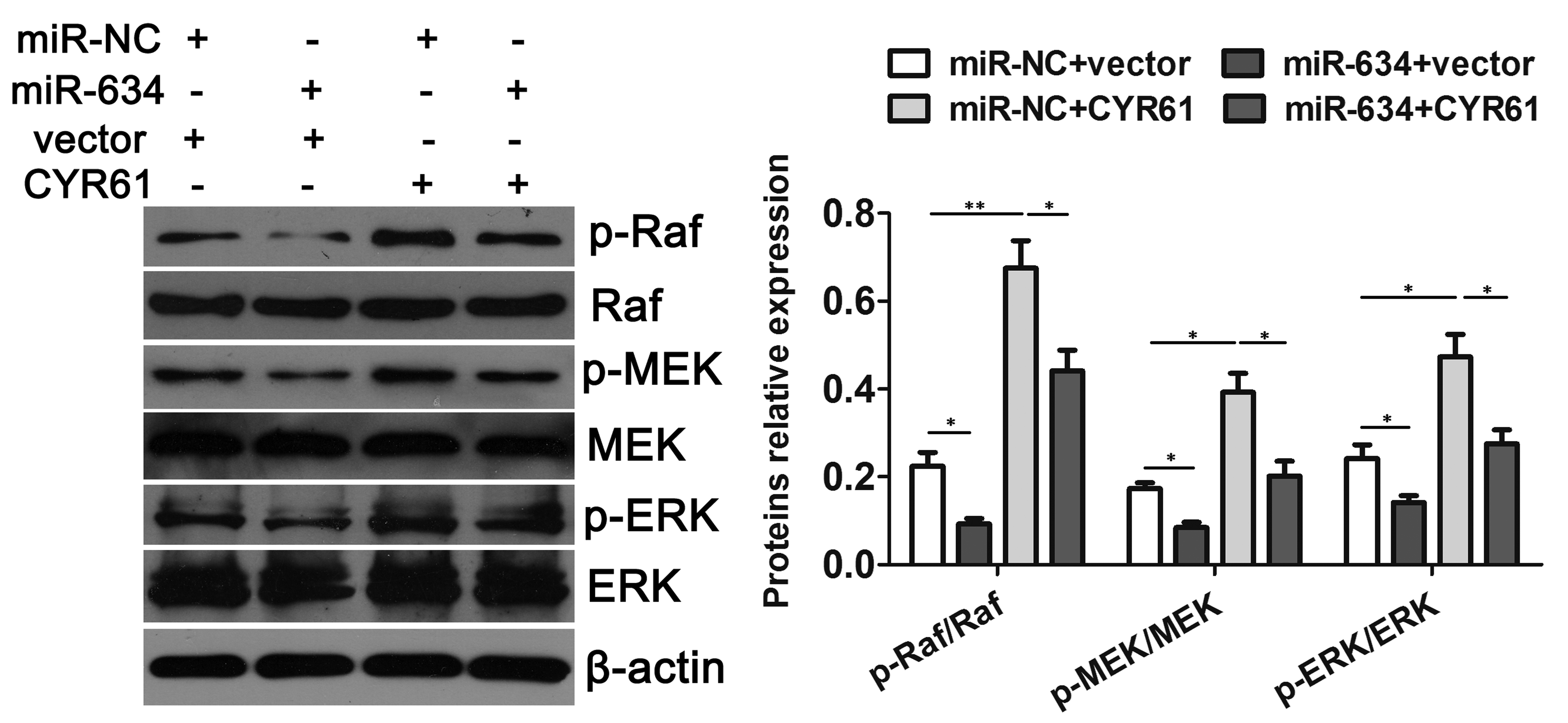

Supplement: Supplementary file 1 — Figure S1. Overexpression of CYR61 reverses the inhibitory effects of miR‐634 downregulation on U87/TMZ cells. [file CAM4-7-913-s001.tif]

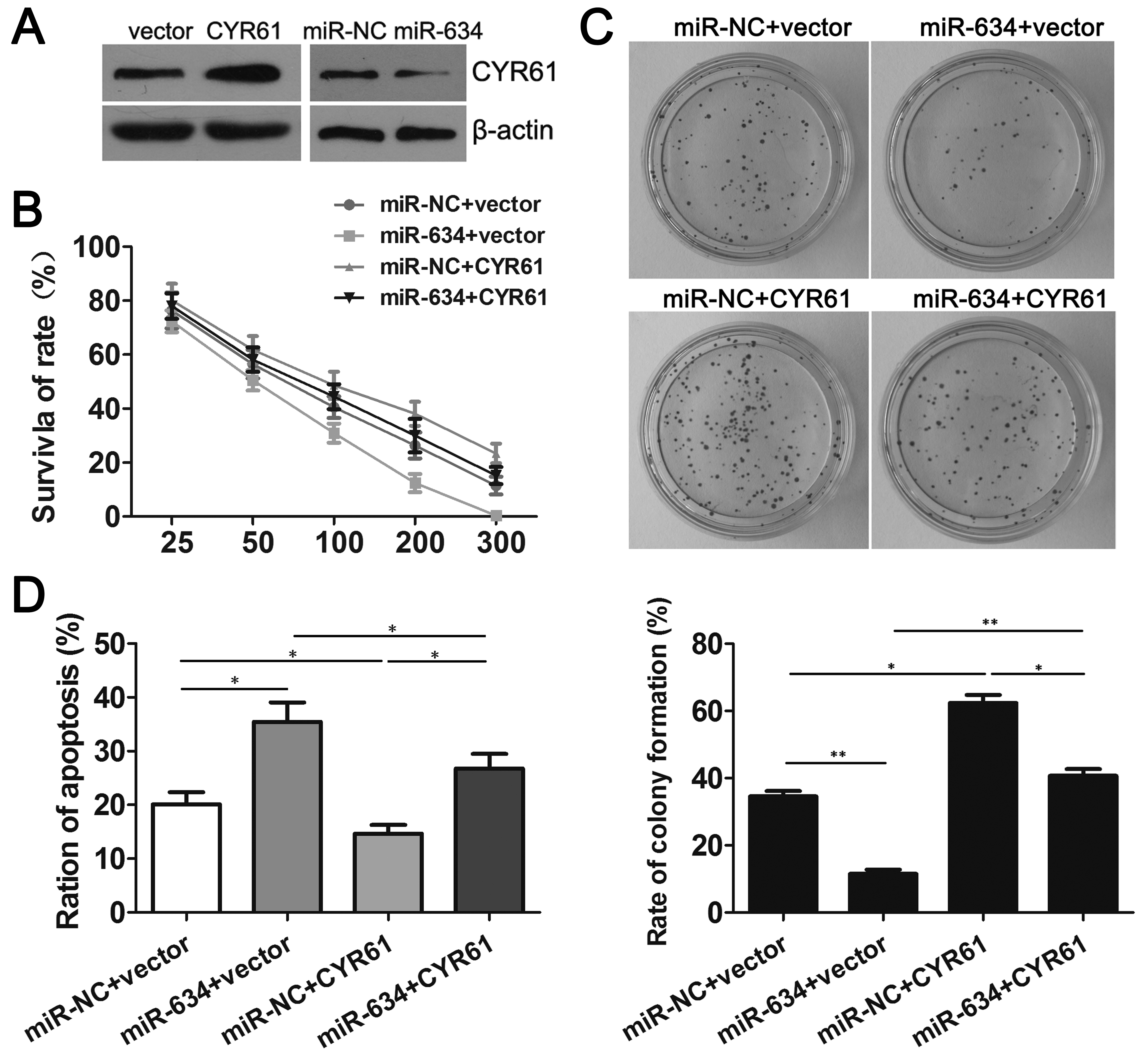

Supplement: Supplementary file 2 — Figure S2. Overexpression of CYR61 reverses the inhibitory effects of miR‐634 downregulation in U87 cells. [file CAM4-7-913-s002.tif]

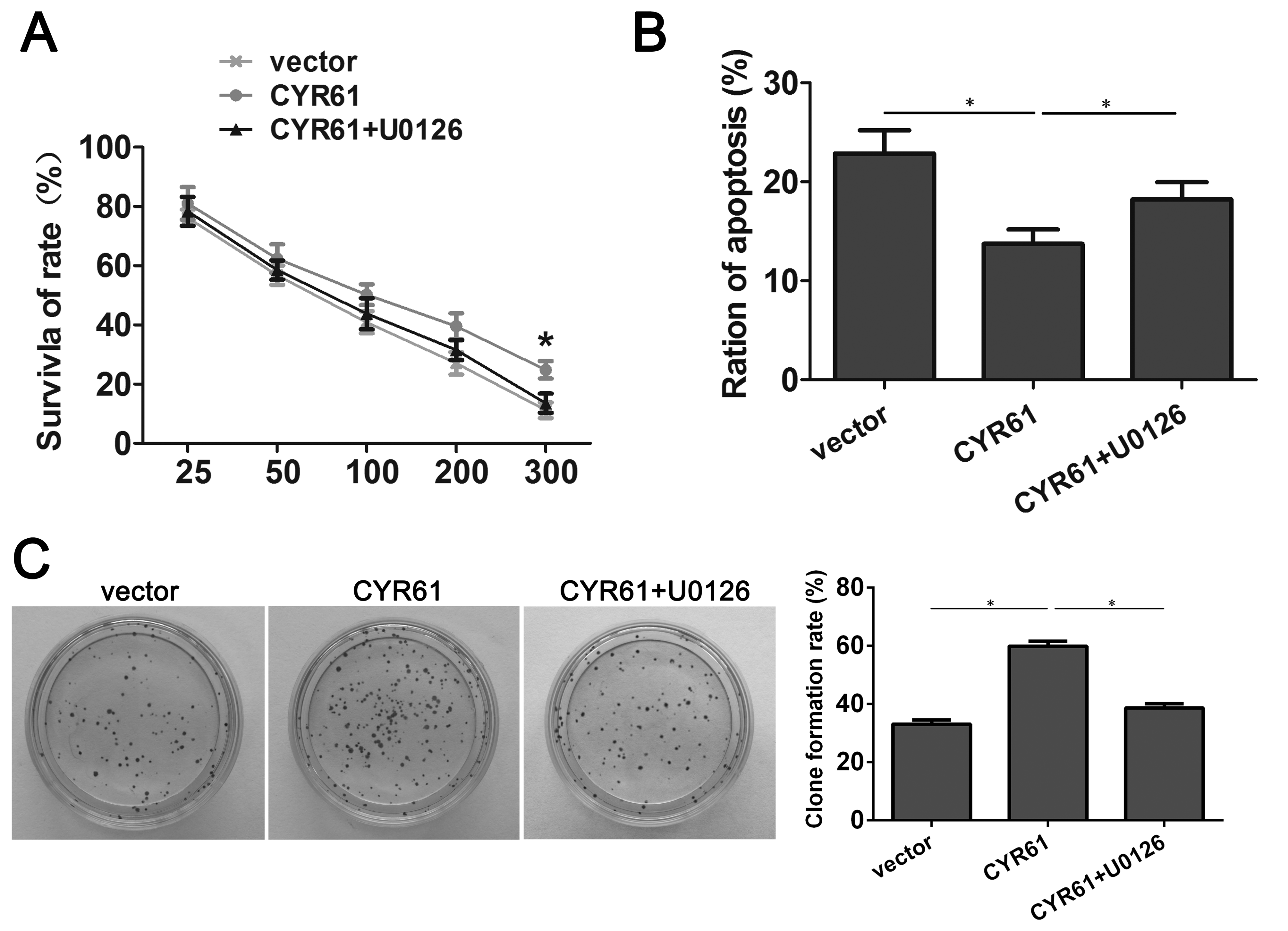

Supplement: Supplementary file 3 — Figure S3. Inactivation of ERK signaling reverses the oncogenic effects of CYR61 in U87 cells. [file CAM4-7-913-s003.tif]
